# Adipose‐Derived Mesenchymal Stem Cells Reduce miR‐214‐5p to Promote Ovarian Cancer Progression via the β‐Catenin Pathway by Targeting PAX8

**DOI:** 10.1155/sci/7152310

**Published:** 2026-08-19

**Authors:** Ningxia Sun, Jing Wang, Hanxu Yang, Xiangyu Liu, Yijing Chu

**Affiliations:** ^1^ Department of Gynecology and Obstetrics, The Affiliated Hospital of Qingdao University, Qingdao University, Qingdao, China, qdu.edu.cn; ^2^ Key Laboratory of Cancer Prevention and Therapy of Tianjin, Department of Gynecologic Oncology, Tianjin Medical University Cancer Institute and Hospital, National Clinical Research Center for Cancer, Tianjin’s Clinical Research Center for Cancer, Tianjin Medical University, Tianjin, China, tmucih.com

**Keywords:** β-catenin, adipose-derived mesenchymal stem cells, miR-214-5p, ovarian cancer, PAX8

## Abstract

**Background:**

Building on our previous work, we found that human omental adipose‐derived mesenchymal stem cells (ADSCs) promote ovarian cancer growth and metastasis by inducing PAX8, a key oncogenic transcription factor in ovarian cancer. TAZ is a core downstream effector of the Hippo signaling pathway, closely associated with malignant progression in multiple tumors, and our prior work confirmed that PAX8 can stabilize TAZ protein. In this study, we aimed to further clarify how ADSCs regulate PAX8 to explore the downstream signaling pathway driving malignant progression in ovarian cancer.

**Materials and Methods:**

Ovarian cancer cells were treated with ADSC‐conditioned medium (ADSC‐CM) to investigate the regulatory effect of ADSCs on miR‐214‐5p expression. qRT‐PCR and western blot were applied to detect the levels of miR‐214‐5p, PAX8, β‐catenin, and TAZ, so as to dissect the regulatory relationship of ADSCs/miR‐214‐5p/PAX8/β‐catenin/TAZ. A dual‐luciferase reporter assay verified the direct targeting interaction between miR‐214‐5p and PAX8. CCK‐8 and Transwell invasion assays were conducted to assess the biological functions of the miR‐214‐5p/PAX8 axis in ovarian cancer progression.

**Results:**

We show that ADSC treatment significantly downregulated miR‐214‐5p in ovarian cancer cells. Moreover, PAX8 is a direct target of miR‐214‐5p: knockdown or overexpression of miR‐214‐5p altered PAX8 expression by directly binding to PAX8 mRNA. Additionally, PAX8 stabilizes TAZ by activating the β‐catenin pathway. Functionally, miR‐214‐5p inhibited ovarian cancer cell proliferation and invasion by downregulating PAX8.

**Conclusion:**

Taken together, ADSCs downregulate miR‐214‐5p to increase PAX8 expression, which sequentially activates the β‐catenin pathway and stabilizes TAZ. This novel signaling axis is responsible for ADSC‐induced ovarian cancer growth and metastasis.

## 1. Introduction

Ovarian cancer is the deadliest gynecologic malignancy [1, 2], and its high mortality and morbidity largely result from a high rate of intraperitoneal dissemination and the development of chemotherapy‐resistant tumors [3, 4], despite the disease’s initial chemosensitivity [5]. The omentum is the primary site of ovarian cancer metastasis. The omental adipose tissue promotes tumor growth and spreads through multiple mechanisms. Numerous studies have examined the role of mesenchymal stem cells (MSCs) in solid tumors [6]. Functionally, MSCs can drive tumor progression directly by altering cancer cell behavior or indirectly by regulating the immune status and angiogenesis. As a subtype of MSCs, adipose‐derived MSCs (ADSCs) can be isolated from human lipoaspirates and exert significant tumor‐promoting functions within the tumor microenvironment. Both ADSCs and bone marrow‐derived MSCs can differentiate into CAFs in a directed manner and continuously secrete immunosuppressive cytokines, including TGF‐β, IL‐6, and IL‐10, to exert immunosuppressive effects [7]. Accumulation of MSCs and ADSCs within tumors neutralizes the anti‐tumor function of complete T cell antigens (CTAs) and drastically impairs the therapeutic response to PD‐1 blockade [8]. In our previous studies, omental ADSCs were shown to promote ovarian cancer growth and metastasis [9]. Moreover, ADSCs can increase PAX8 expression in ovarian cancer cells and promote proliferation. TAZ (transcriptional coactivator with PDZ‐binding motif, also known as WWTR1), the core downstream effector of the Hippo signaling cascade, interacts with PAX8 to maintain its protein stability and mediate oncogenic signals. Therefore, in the present study, we aimed to further clarify the mechanism of PAX8 in ovarian cancer cells.

PAX8 is a member of the paired box (PAX) gene family. It encodes a protein with an evolutionarily conserved paired‐box DNA‐binding domain [10]. PAX8 is essential for determining cell fate during organ and tissue development; in adult normal tissues, it is expressed in the kidney, thyroid, and fallopian tubes but not in the ovarian epithelium [11]. Staining showed a significant increase in PAX8 expression in ovarian and endometrial cancer tissues compared with adjacent tissues, particularly in serous ovarian tumors and in clear cell carcinoma [12, 13]. Moreover, ovarian cancer patients with high PAX8 levels are more likely to relapse and generally have a worse clinical prognosis [14]. Given the reported oncogenic role of PAX8 in ovarian cancer, we next sought to identify upstream microRNAs (miRNAs) that may negatively regulate PAX8. Through bioinformatic prediction analyses, we speculated that PAX8 could serve as a downstream target of miR‐214‐5p, and it has been shown to play a key role in multiple cancers. In ovarian cancer, it acts as a tumor suppressor and is expressed at low levels [15], in cervical cancer [16, 17], and lung cancer [18]. However, miR‐214‐5p can also be used as a cancer promoter, which has been reported to be low‐expressed in gastric cancer [19] and pancreatic cancer [20]. Subsequently, we conducted experiments to further elucidate the role and mechanism of miR‐214‐5p–mediated regulation of PAX8 in ovarian cancer cells.

Building on our prior work, we found that ADSC‐induced upregulation of PAX8 may promote ovarian cancer cell proliferation by regulating TAZ stability. Here, we further explore the role and mechanism of PAX8 in ovarian cancer using SKOV3 and A2780 cells as models. Our findings indicate that miR‐214‐5p may inhibit tumor progression by downregulating PAX8, thereby impairing TAZ stability partially through the Wnt/β‐catenin pathway in ovarian cancer cells.

## 2. Materials and Methods

### 2.1. Cell Culture

The human ovarian cancer cell lines SKOV3 and A2780 were obtained from the Type Culture Collection China Centre. Cells were cultured in DMEM/F12 (Gibco, USA) supplemented with 10% fetal bovine serum and 1% penicillin/streptomycin and maintained at 37°C in a 5% CO_2_ incubator. Preparation of conditioned medium and identification of ADSCs were performed as previously described [21]. Briefly, when ADSCs reached ~80% confluence, the medium was replaced with serum‐free DMEM/F12 for 24 h; the collected medium was then centrifuged and filtered through 0.22‐µm filters (Millipore, USA).

### 2.2. Cell Transfection

Two ovarian cancer cell lines were cultured in 24‐well or 12‐well plates for 24 h until the cell aggregation rate reached more than 70%–80%. Then, proper lentivirus and siRNAs were transfected into cells, respectively, using Lipofectamine 2000 (Invitrogen, USA). miR‐214‐5p mimics and miR‐214‐5p inhibitors were synthesized by Hanbio (China). The sequences of the siRNAs used are shown in Table 1.

**Table 1:** The sequences of the siRNAs.

PAX8	5′‐UCUUUAUUUAUUACAUGAA‐3′
Nontarget	5′‐GCAAGCTGACCCTGAAGTT‐3
β‐Catenin	5′‐CCCACTAATGTCCAGCGTT‐3
miR‐214‐5p mimics	AGAGUUGUCAUGUGUCU
miR‐214‐5p inhibitor	AGACACAUGACAACUCU

### 2.3. Cell Proliferation

Transfected cells (5 × 10^3^/well) were seeded in a 96‐well plate and incubated for 24, 48, and 72 h in a 37°C incubator with a 5% CO_2_ atmosphere. Each well was added with 10 μL CCK‐8 reagent (Thermo Fisher Scientific, USA) and cultured for an extra 1.5 h before measuring the optical density (OD) of each well at a wavelength of 450 nm using a microplate reader.

### 2.4. Cell Invasion

Transfected cells (2.5 × 10^4^ per well) suspended in serum‐free medium were seeded into the upper chambers of Transwell inserts (pore membranes, 8 μm). The lower chambers contained a complete medium with 10% FBS. Inserts were precoated with or without Matrigel (Invitrogen). After 24 h of incubation at 37°C with 5% CO_2_, cells were fixed in 4% paraformaldehyde, washed with PBS, and stained with 1% crystal violet. Cells remaining on the upper surface of the membrane were gently removed with cotton swabs. Images were acquired using an inverted light microscope. Each experiment was performed at least three times.

### 2.5. RNA Isolation and Real‐Time Quantitative PCR (RT‐qPCR)

Total RNA was extracted from ovarian cells using TRIzol reagent (Takara, Japan). cDNA was synthesized with reverse transcription kits (Invitrogen). qRT‐PCR was performed using the SYBR Premix Ex Taq kit (Takara, Japan) on an Applied Biosystems 7500 Sequence Detection System. GAPDH served as the internal control. All reactions were run in triplicate. The primer sequences used for qRT‐PCR are as shown in Table 2.

**Table 2:** The primer sequences used for qRT‐PCR.

PAX8	Forward: 5′‐GATCCTCACTCACCCTTCGC‐3′
	Reverse: 5′‐TAACCACACAGGGAGTGTGC‐3′
miR‐214‐5p	Forward: 5′‐GTATCTGTCTATGAGCAAAGGAAACC‐3′
	Reverse: 5′‐GGTGTAGATGCTATGGTGTGAGGGC‐3′
GAPDH	Forward: 5′‐AATGGGCAGCCGTTAGGAAA‐3′
	Reverse: 5′‐GCGCCCAATACGACCAAATC‐3′

### 2.6. Western Blotting

Cells were lysed in RIPA buffer supplemented with phenylmethanesulfonyl fluoride (PMSF, 1:100). Protein concentrations were determined using the BCA Protein Assay Kit (Beyotime, China). Equal amounts of protein were separated by SDS–PAGE and transferred to PVDF membranes (Bio‐Rad, USA). After blocking with 10% nonfat dry milk in TBST (Solarbio, China), membranes were incubated overnight at 4°C with diluted primary antibodies and then with goat anti‐rabbit IgG for 2 h at room temperature. Signals were detected by chemiluminescence (Bio‐Rad, USA) and quantified using ImageJ. Primary antibodies: PAX8 (ab53490, 1:1000, Abcam, UK), TAZ (ab205270, 1:1000, Abcam, UK), c‐MYC (ab32072, 1:1000, Abcam, UK), Snail (ab229701, 1:1000, Abcam, UK), β‐catenin (#8480, 1:1000, Cell Signaling Technology, USA), and GAPDH (ab181603, 1:5000, Abcam, UK).

### 2.7. Luciferase Reporter

Cells with a good growth index were evenly seeded into 96‐well plates. The miR‐214‐5p mimic and PAX8 wild‐type (WT) or mutant (MUT) dual‐luciferase reporter vectors were cotransfected into the stably infected cells using Lipofectamine 3000 (Thermo Fisher Scientific, USA). miR‐NC served as the control. At 24 h posttransfection, luciferase activity was measured using the Dual‐Luciferase Reporter Assay Kit (Promega, USA).

### 2.8. Statistical Analysis

Data were analyzed in GraphPad Prism 8.4.3. Comparisons between two groups used two‐tailed Student’s *t*‐tests, and multiple groups were compared by one‐way ANOVA. Values are presented as the mean ± standard error of the mean (SEM) from at least three independent experiments. *p* < 0.05 ( ^∗^) and *p* < 0.01 ( ^∗∗^) were considered statistically significant.

## 3. Results

### 3.1. ADSCs Reduce miR‐214‐5p Expression in Ovarian Cancer Cells

**Figure 1:**
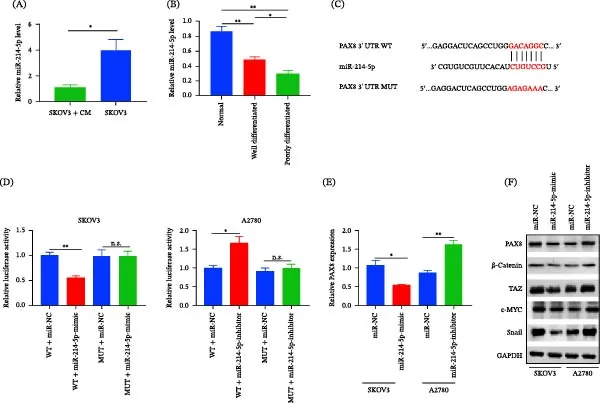
miR‐214‐5p was induced by ADSCs and directly targets PAX8 mRNA 3′ UTR and regulates PAX8 expression in ovarian cancer cells. (A) qPCR was adopted to evaluate the miR‐214‐5p level in SKOV3 cells with or without ADSC CM. (B) qPCR was adopted to evaluate the miR‐214‐5p level in various differentiated tissue types. (C) Mutant and binding sites between miR‑214‑5p and PAX8. (D) Luciferase activity in SKOV3 and A2780 cells cotransfected with miR‑214‑5p/miR‑NC mimics, miR‑214‑5p/miR‑NC inhibitors, and PAX8‑WT or PAX8‑MUT reporter plasmids. (E) qPCR was adopted to evaluate the PAX8 mRNA expression levels in ovarian cancer cells when cotransfected with miR‑214‑5p mimics and miR‑214‑5p inhibitors. (F) PAX8, TAZ, and β‐catenin signaling pathway gene expression levels in ovarian cancer cells when cotransfected with miR‑214‑5p mimics and miR‑214‑5p inhibitors. Quantitative statistical analysis of WB is shown in Figure S1. All experiments were independently repeated three times. Statistical analysis was conducted via two‐tailed Student’s *t*‐test and one‐way ANOVA. The results are presented as mean ± standard error of the mean (SEM).  ^∗^ *p* < 0.05 and  ^∗∗^ *p* < 0.01 were considered statistically significant.

qPCR was used to evaluate miR‐214‐5p levels in SKOV3 cells with or without ADSC‐conditioned medium (ADSC‐CM). The results showed that miR‐214‐5p in SKOV3 cells treated with ADSC‐CM was lower than that in SKOV3 cells cultured alone (Figure 1A). In our further analysis, we found that miR‐214‐5p expression was lowest in poorly differentiated epithelial ovarian cancer tissues (Figure 1B).

### 3.2. miR‐214‐5p Direct Targets PAX8 mRNA 3′ UTR and Regulates PAX8 Expression

During target prediction, we identified a putative miR‐214‐5p binding site in the PAX8 3′‐UTR; the WT and MUT sequences are shown in Figure 1C. Our previous work confirmed that SKOV3 cells express higher PAX8 levels, whereas A2780 cells express relatively lower levels [9]. We cotransfected SKOV3 cells with miR‐214‐5p mimics and either wt‐PAX8‐3′ UTR or mut‐PAX8‐3′ UTR reporters and cotransfected A2780 cells with miR‐214‐5p inhibitors and the same reporters. Luciferase assays showed that in SKOV3 cells, the WT reporter activity was reduced, whereas in A2780 cells, it was increased; in both cell lines, the MUT reporter was unchanged (Figure 1D). Separately, we transfected SKOV3 cells with miR‐214‐5p mimics and A2780 cells with miR‐214‐5p inhibitors. RT‐qPCR and western blotting showed decreased PAX8 in SKOV3 and increased PAX8 in A2780 relative to the NC group (Figure 1E, F). Together, these findings indicate that miR‐214‐5p regulates PAX8 expression in ovarian cancer cells.

### 3.3. PAX8 Altered Expression Counteracts Effects of miR‐214‐5p on Cell Proliferation, Migration, and Invasion in Ovarian Cancer Cells

**Figure 2:**
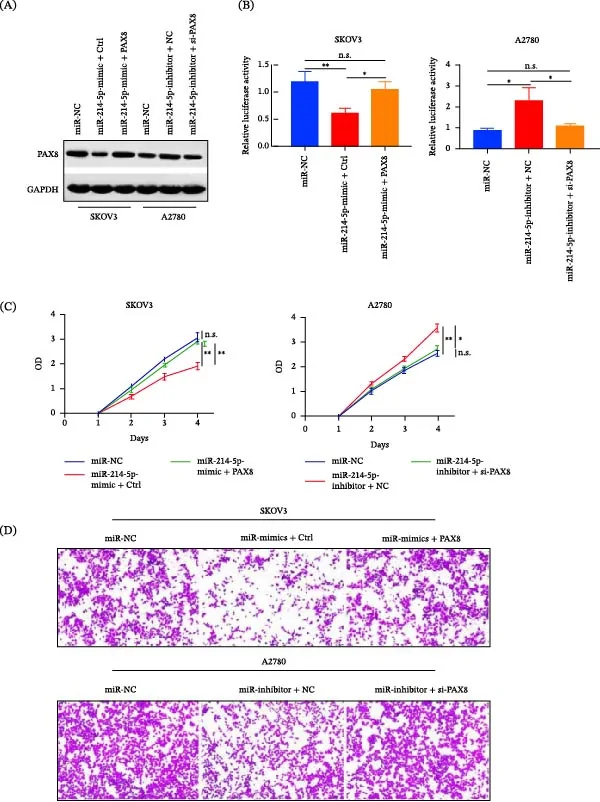
PAX8 altered expression counteracts the effects of miR‐214‐5p on cell proliferation, migration, and invasion in ovarian cancer cells. (A) PAX8 protein expression levels in ovarian cancer cells when cotransfected with miR‑214‑5p mimics, miR‑214‑5p inhibitors, OE‐PAX8, and si‐PAX8. Quantitative statistical analysis of WB is shown in Figure S2. (B) Luciferase reporter activity was detected in ovarian cancer cells when cotransfected with miR‑214‑5p mimics, miR‑214‑5p inhibitors, OE‐PAX8, and si‐PAX8. Cell proliferation (C) and invasion ability (D) were measured in SKOV3 and A2780 cells after miR‐214‐5p mimics, miR‐214‐5p inhibitor, OE‐PAX8, si‐PAX8 transfection by CCK8 assay and Transwell invasion assay. All experiments were repeated three times independently. Statistical analysis was conducted via two‐tailed Student’s *t*‐test and one‐way ANOVA. The results are presented as mean ± standard error of the mean (SEM).  ^∗^ *p* < 0.05 and  ^∗∗^ *p* < 0.01 were considered statistically significant.

To further assess the role of the miR‐214‐5p/PAX8 axis in ovarian cancer, we performed gain‐ and loss‐of‐function experiments in SKOV3 and A2780 cells. Cells were transfected with miR‐214‐5p mimics, miR‐214‐5p inhibitor, PAX8, or si‐PAX8, alone or in combination. Western blotting showed that miR‐214‐5p negatively regulates PAX8, and coexpression of PAX8 partially rescued the miR‐214‐5p–mediated suppression (Figure 2A, B). In proliferation assays (Figure 2C), miR‐214‐5p inhibited cell growth, whereas PAX8 promoted growth and counteracted the inhibition by miR‐214‐5p; conversely, si‐PAX8 suppressed growth and reversed the stimulation by the miR‐214‐5p inhibitor. Transwell assays (Figure 2D) showed that miR‐214‐5p reduced migration and invasion, and these effects were reversed by PAX8. Overall, miR‐214‐5p negatively regulates the PAX8 expression and function in ovarian cancer cells.

### 3.4. TAZ Was Upregulated in Ovarian Cancer Cells and Was Regulated by PAX8 Through β‐Catenin Pathway

**Figure 3:**
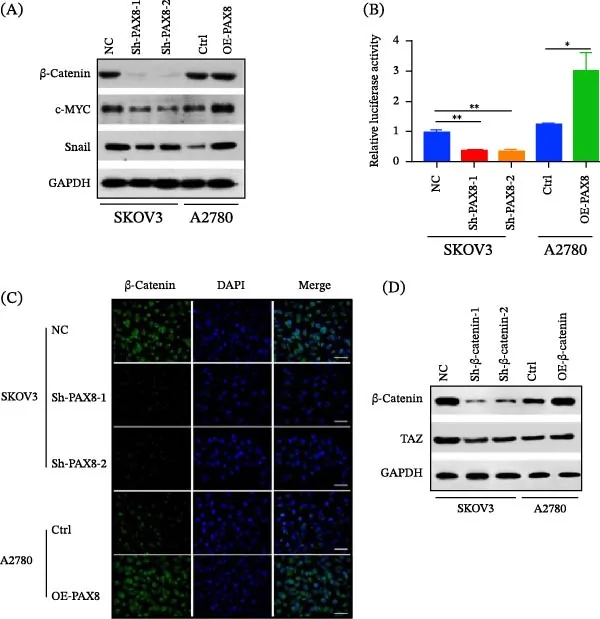
TAZ was upregulated in ovarian cancer cells and was regulated by PAX8 through β‐catenin pathway. (A) β‐Catenin protein expression levels in ovarian cancer cells when cotransfected with OE‐PAX8 and si‐PAX8. (B) Luciferase reporter activity was detected in ovarian cancer cells when cotransfected with OE‐PAX8 and si‐PAX8. (C) Immunofluorescence assay in ovarian cancer cells when cotransfected with OE‐PAX8 and si‐PAX8. (D) TAZ protein expression levels in ovarian cancer cells when cotransfected with OE‐β‐catenin and si‐β‐catenin. Quantitative statistical analysis of WB is shown in Figure S3. All experiments were repeated three times independently. Statistical analysis was conducted via two‐tailed Student’s *t*‐test and one‐way ANOVA. The results are presented as mean ± standard error of the mean (SEM).  ^∗^ *p* < 0.05 and  ^∗∗^ *p* < 0.01 were considered statistically significant.

Our previous study showed that PAX8 increases TAZ expression by stabilizing the TAZ protein. To further define PAX8’s role in ovarian cancer, we examined β‐catenin and its target genes (c‐MYC and Snail) in cells transfected with PAX8 siRNA or a PAX8 overexpression plasmid. PAX8 knockdown significantly reduced β‐catenin, c‐MYC, and Snail in SKOV3 cells, whereas PAX8 overexpression increased these proteins in A2780 cells (Figure 3A). We also confirmed that PAX8 influences β‐catenin signaling using luciferase reporter assays and immunofluorescence in both cell lines (Figure 3B, C). Next, we transfected si‐β‐catenin into SKOV3 cells and a β‐catenin overexpression plasmid into A2780 cells and found that TAZ protein levels decreased in SKOV3 but increased in A2780 (Figure 3D). In summary, PAX8 elevates TAZ via the Wnt/β‐catenin pathway, acting on β‐catenin signaling to regulate TAZ expression.

## 4. Discussion

### 4.1. Results in the Context of Published Literature

ADSCs are components of the tumor microenvironment and can be isolated from human lipoaspirates. Our previous study showed that omental ADSCs promote ovarian cancer cell proliferation and invasion and contribute to the formation of premetastatic niches in the omentum [21]. Besides, our results confirmed that ADSCs upregulated PAX8 expression to stabilize TAZ proteins and promoted its transcription in ovarian cancer.

Emerging evidence indicates that miRNAs participate in the posttranscriptional regulation of target gene expression. Aberrant expression of miRNAs is closely associated with the progression and chemoresistance of malignancies [22–25]. Mechanistically, miRNAs interact with core tumor‐suppressive genes [23] and remodel cellular energy metabolism [24], thereby driving cancer metastasis and therapeutic resistance. miR‐214 is a multifunctional miRNA with context‐dependent roles in human malignancies [26, 27]. For example, miR‐214‐5p suppresses proliferation, migration, and invasion by targeting SEMA4C and SOX4 in cervical cancer cells [16, 17]. However, some studies have reported upregulation of miR‐214‐5p in certain cancers, including glioma tissues and a cisplatin‐resistant tongue squamous cell carcinoma subline [28, 29]. Growing evidence indicates that miR‐214‐5p acts as a tumor suppressor in ovarian cancer [26].

Accumulating evidence has indicated that PAX8 is aberrantly expressed in multiple solid tumors, where it regulates tumor cell proliferation, differentiation, and metastasis [30–32]. Hu et al. [33] reported that PAX8 overexpression in endometrial cancer is associated with poor prognosis based on R‐based analyses and immunohistochemistry. Conversely, patients with low PAX8 expression and CpG island hypermethylation had better outcomes, supporting PAX8 methylation as a potential marker for early diagnosis and prognosis [33]. PAX8 is upregulated in epithelial ovarian cancer. Curcumol inhibits ovarian cancer by suppressing PAX8 expression, thereby blocking multiple tumorigenic processes—including proliferation, migration, invasion, angiogenesis, and the growth of ovarian cancer xenografts in nude mice—and it shows a synergistic effect with niraparib [34]. In our previous study, PAX8 functioned as an oncogenic factor regulating cancer progression. ADSCs increased ovarian cancer cell proliferation by upregulating PAX8 and stabilizing the TAZ protein in ovarian cancer cells [9].

β‐Catenin is a key regulator in the canonical Wnt pathway [35], controlling tumor initiation, growth, and metastasis [36]. Multiple studies have shown that noncoding RNAs contribute to ovarian cancer progression by modulating Wnt/β‐catenin signaling [37, 38]. Downregulation of β‐catenin reduces the proliferation, migration, and invasion of OC cells. CTNNB1 encodes the functional protein β‐catenin. Bioinformatic analyses indicate that miR‐214‐5p and CTNNB1 have multiple putative binding sites, suggesting that miR‐214‐5p may directly target CTNNB1. Zhou et al. [39] showed by molecular experiments that C3G downregulates β‐catenin expression via miR‐214‐5p in LN‐18/TR cells.

### 4.2. Summary of Main Results

Although how ADSCs mediate TAZ stability through PAX8 in ovarian cancer cells was unclear, we investigated these interactions in the current study. Bioinformatic analyses indicated potential binding sites for miR‐214‐5p in PAX8; dual‐luciferase reporter assays further suggested a potential direct interaction between miR‐214‐5p and PAX8 in vitro. We next performed transfections in SKOV3 and A2780 cells. The effects of miR‐214‐5p on cell proliferation, migration, and invasion were largely neutralized by manipulating PAX8 levels, indicating that PAX8 mediates part of the tumor‐suppressive functions. To elucidate the interplay among miR‐214‐5p, PAX8, and β‐catenin, we conducted additional cell and molecular experiments. In SKOV3 cells cocultured with ADSC‐CM, miR‐214‐5p levels were significantly higher than in SKOV3 cells cultured alone. Nevertheless, miR‐214‐5p markedly inhibited proliferation, migration, and invasion, which may reflect the dominant effects of factors present in ADSC CM under these conditions. In clinical samples, miR‐214‐5p expression was lower in epithelial ovarian cancer tissues than in higher differentiation levels, suggesting a link with prognosis. However, the precise mechanisms by which ADSCs contribute to TAZ stability via PAX8 remain to be fully clarified. Further results showed that β‐catenin and its target genes (c‐MYC and Snail) were downregulated after PAX8 knockdown in ovarian cancer cells. For additional verification, we transfected SKOV3 cells with si‐β‐catenin and A2780 cells with a β‐catenin overexpression plasmid; TAZ protein levels decreased in SKOV3 and increased in A2780, varying positively with β‐catenin expression. In sum, PAX8 acts on the β‐catenin signaling pathway to regulate TAZ expression, providing a mechanistic link among miR‐214‐5p, PAX8, and β‐catenin in ovarian cancer cells.

### 4.3. Strengths and Weaknesses

The novelty of this study lies in its focus on the mechanisms of ovarian cancer metastasis. However, several limitations should be acknowledged. First, although dual‐luciferase assays support the in vitro binding between miR‐214‐5p and PAX8, further validation is required to consolidate the endogenous targeting relationship. Second, further validation in clinical specimens is needed; these analyses will be conducted in future work.

### 4.4. Implications for Practice and Future Research

PAX8 may regulate cancer progression, including ADSC‐induced tumor development, and it helps modulate interactions between the tumor microenvironment and primary tumors. Our study provides insights for the future development of ovarian cancer–specific preventive therapy.

## 5. Conclusion

In conclusion, ADSCs promote tumor progression at least partially by upregulating PAX8 expression, which is related to the downregulation of miR‐214‐5p. PAX8 upregulates β‐catenin expression, which stabilizes TAZ in ovarian cancer cells. Therefore, PAX8 may be a potential therapeutic target for ovarian cancer.

## Author Contributions

**Ningxia Sun**: investigation, writing – original draft. **Jing Wang**: supervision, writing – review and editing. **Hanxu Yang**: resources, investigation. **Xiangyu Liu and Yijing Chu**: writing – review and editing.

## Funding

The authors have nothing to report.

## Disclosure

PAX8 can potentially regulate cancer progression, that is ADSC‐induced tumor development, and participated in modulating the interaction between TME and primary tumors. Our study provides insights into developing ovarian cancer‐specific preventive therapy in the future. All authors have consented to the publication of this article.

## Ethics Statement

The authors have nothing to report.

## Conflicts of Interest

The authors declare no conflicts of interest.

## Supporting Information

Additional supporting information can be found online in the Supporting Information section.

## Supporting information


**Supporting Information** Figure S1: Quantitative statistical analysis of PAX8, TAZ, and β‐catenin signaling pathway gene expression levels in ovarian cancer cells when cotransfected with miR‑214‑5p mimics and miR‑214‑5p inhibitors. Relative protein levels were normalized to the internal reference GAPDH. Scatter points represent individual biological replicates. Statistical analysis was performed via Student’s *t*‐test. Data are expressed as mean ± SD.  ^∗^ *p* < 0.05,  ^∗∗^ *p* < 0.01, and  ^∗∗∗^ *p* < 0.001. Figure S2: Quantitative statistical analysis of PAX8 levels determined by western blot in ovarian cancer cells when cotransfected with miR‑214‑5p mimics, miR‑214‑5p inhibitors, OE‐PAX8, and si‐PAX8. Relative protein levels were normalized to the internal reference GAPDH. Scatter points represent individual biological replicates. Statistical analysis was performed via one‐way ANOVA. Data are expressed as mean ± SD.  ^∗^ *p* < 0.05,  ^∗∗^ *p* < 0.01, and  ^∗∗∗^ *p* < 0.001. Figure S3: (a) Quantitative statistical analysis of β‐catenin protein expression levels in ovarian cancer cells when cotransfected with OE‐PAX8 and si‐PAX8. (b) Quantitative statistical analysis of TAZ protein expression levels in ovarian cancer cells when cotransfected with OE‐β‐catenin and si‐β‐catenin. Relative protein levels were normalized to the internal reference GAPDH. Scatter points represent individual biological replicates. Statistical analysis and one‐way ANOVA were performed via Student’s *t*‐test. Data are expressed as mean ± SD.  ^∗^ *p* < 0.05,  ^∗∗^ *p* < 0.01, and  ^∗∗∗^ *p* < 0.001.

## Data Availability

All data are available from the corresponding author upon reasonable request.
