# Supplementary material for: Adipose‐Derived Mesenchymal Stem Cells Reduce miR‐214‐5p to Promote Ovarian Cancer Progression via the β‐Catenin Pathway by Targeting PAX8
Source: Stem Cells Int. 2026 Aug 19;2026:7152310. doi: 10.1155/sci/7152310 (PMC13490915; doi:10.1155/sci/7152310)
Supplement: Supplementary file 1 [file SCI-2026-7152310-s001.docx]

Supplementary Figure S1. Quantitative statistical analysis of PAX8, TAZ, and β-catenin signaling pathway gene expression levels in ovarian cancer cells when co‑transfected with miR‑214‑5p mimics and miR‑214‑5p inhibitors. Relative protein levels were normalized to the internal reference GAPDH. Scatter points represent individual biological replicates. Statistical analysis was performed via Student’s t-test. Data are expressed as mean ± SD. **P* < 0.05, ***P* < 0.01, ****P* < 0.001.

Supplementary Figure S2. Quantitative statistical analysis of PAX8 levels determined by western blot in ovarian cancer cells when co‑transfected with miR‑214‑5p mimics, miR‑214‑5p inhibitors, OE-PAX8, and siPAX8. Relative protein levels were normalized to the internal reference GAPDH. Scatter points represent individual biological replicates. Statistical analysis was performed via one-way ANOVA. Data are expressed as mean ± SD. **P* < 0.05, ***P* < 0.01, ****P* < 0.001.

a .

b .

Supplementary Figure S3. (a). Quantitative statistical analysis of β-catenin protein expression levels in ovarian cancer cells when co‑transfected with OE-PAX8 and si-PAX8. (b). Quantitative statistical analysis of TAZ protein expression levels in ovarian cancer cells when co‑transfected with OE-β-catenin and si-β-catenin. Relative protein levels were normalized to the internal reference GAPDH. Scatter points represent individual biological replicates. Statistical analysis and one-way ANOVA were performed via Student’s t-test. Data are expressed as mean ± SD. **P* < 0.05, ***P* < 0.01, ****P* < 0.001.
